# Enhancing functional motor outcomes in chronic stroke: A systematic review of the growing role of non-invasive brain stimulation

**DOI:** 10.1371/journal.pone.0327583

**Published:** 2025-07-28

**Authors:** Mirjam Bonanno, Maria Grazia Maggio, Angelo Quartarone, Giovanni Morone, Alessandro Marco De Nunzio, David Militi, Carmela Casella, Svonko Galasso, Rocco Salvatore Calabrò

**Affiliations:** 1 IRCCS Centro Neurolesi Bonino Pulejo, Messina, Italy; 2 Department of Life, Health and Environmental Sciences, University of L’Aquila, L’Aquila, Italy; 3 IRCCS Santa Lucia foundation, Rome, Italy; 4 Department of Research and Development, LUNEX International University of Health, Differdange, Luxembourg; 5 Odontostomatology and Dental Surgery study, Messina, Italy; 6 AOU Policlinico G. Martino, Messina, Italy; University of Zanjan, IRAN, ISLAMIC REPUBLIC OF

## Abstract

**Introduction:**

Non-invasive brain stimulation (NIBS) promotes functional recovery by enhancing neuroplasticity and reorganizing brain networks. It is hypothesized that transcranial magnetic stimulation (TMS), repetitive transcranial magnetic stimulation (rTMS), or intermittent θ-burst stimulation (i-TBS) as well as trans direct current stimulation (tDCS) can modulate synaptic connectivity, through magnetic or electric stimuli, directly on the brain area. This systematic review aims to address the lack of comprehensive syntheses focusing on the comparative effectiveness of NIBS techniques, including tDCS, rTMS, and iTBS, on distinct motor domains (upper limb, lower limb, and vocal/swallowing functions) in chronic stroke patients.

**Evidence acquisition:**

A systematic search was conducted for all peer-reviewed articles published from January 2010 through September 2023, using the following databases: PubMed, Embase, Cochrane Database of Systematic Reviews, PEDro, RehabData, and Web of Science. This systematic review was performed following the recommendations of the Preferred Reporting Items for Systematic Reviews and Meta-Analyses (PRISMA) guidelines, with a registration number in the Prospective Register of Systematic Reviews (PROSPERO 2023) CRD42023458370. A total of 58 studies were included in the qualitative synthesis: 27 focused on upper limb rehabilitation, 13 on lower limb function, 13 on the combination of NIBS and robotic therapy, and 6 on vocal or swallowing functions. Most studies (78%) were randomized controlled trials. Among the NIBS techniques, tDCS showed stronger evidence for gait and balance recovery, while rTMS appeared more effective for upper limb function. Overall, the majority of studies presented a low risk of bias, although methodological issues such as inadequate randomization or blinding affected the reliability of some findings.

**Conclusion:**

We have systematically reviewed the literature on the use of NIBS to enhance motor outcomes (upper limb, lower limb, and vocal/swallowing functions) in individuals with chronic stroke. Findings indicate that rTMS demonstrates greater efficacy in improving upper limb motor function, whereas tDCS appears to be more effective in enhancing gait and balance recover.

## Introduction

Non-invasive brain stimulation (NIBS) is a promising approach to promote functional recovery after neurological injury, such as cerebro-vascular impairments, by modulating cortical excitability and influencing neuroplastic processes [1,2]. NIBS includes two main categories: i) transcranial magnetic stimulation (TMS) with different protocols and applications, e.g., repetitive TMS (rTMS); intermittent theta-burst stimulation (iTBS); and ii) transcranial electrical stimulation (tES), including transcranial direct current stimulation (tDCS); transcranial alternating current stimulation (tACS) and transcranial random noise stimulation (tRNS) [3]. In detail, TMS delivers magnetic pulses to specific brain regions, inducing changes in neuronal activity. rTMS, the most widely used form in clinical practice [4], can exert either inhibitory effects at low frequencies (≤1 Hz) or excitatory effects at higher frequencies (≥5 Hz) [5]. iTBS delivers rapid bursts of high-frequency stimulation (typically at 50 Hz) in patterned intervals. This TMS protocol offers the advantage of shorter treatment sessions, enhancing clinical feasibility, especially for the management of psychiatric symptoms (e.g., drug-resistant depression), compared to standard rTMS [6–9]. In contrast, tES uses weak electrical currents, typically not perceived by the patient, to alter cortical excitability. Indeed, it applies current through electrodes placed on the scalp, generally guided by the EEG 10–20 system [10,11]. Depending on the polarity, tDCS can induce either hyperpolarisation (cathodal, inhibitory) or depolarization (anodal, excitatory) of neuronal membranes, thereby modulating spontaneous neural activity [12,13]. These stimulation techniques are increasingly being explored in post-stroke rehabilitation [14], where modulation of maladaptive plasticity and interhemispheric imbalance is a key therapeutic target [13,15,16]. For instance, following cerebrovascular impairments like stroke, increased excitability of the contralesional hemisphere may suppress activity in the affected hemisphere [16]. By affecting these neural dynamics, NIBS can potentially enhance motor and cognitive improvement in stroke patients [17]. On a brain level, NIBS can modulate the excitability of the cortex and re-balance disrupted interhemispheric communication that normally occurs post-stroke [1]. For instance, procedures such as TMS or tDCS potentially enhance activity within lesioned or underactive areas or suppress maladaptive hyperactivity in contralesional areas. Such adaptations would promote improved communication between neurons, enable synaptic plasticity, and assist in reorganization of functionally important networks required for recovery [1,2]. While NIBS has been widely applied across different stages of stroke recovery, patients in the chronic phase represent a particularly compelling subgroup for targeted interventions. In the chronic stage, spontaneous neurological recovery has plateaued, and functional improvements depend more heavily on external modulation strategies such as neurostimulation [18,19]. Furthermore, the neuroplastic mechanisms engaged in the chronic phase differ from those in acute and subacute stages, potentially requiring different stimulation parameters and therapeutic approaches [18,19]. Despite the growing interest in NIBS, previous reviews have often approached this topic with broad inclusion criteria, including mixed stroke phases and intervention types, or focused on specific motor functions [20–24].

As a result, direct comparisons between specific NIBS modalities (e.g., tDCS, rTMS, iTBS) concerning targeted motor domains remain limited. Furthermore, the literature exploring the integration of NIBS with additional rehabilitation strategies, such as robotic therapy, is still emerging and lacks a systematic synthesis. This systematic review aims to address the lack of comprehensive syntheses focusing on the comparative effectiveness of NIBS techniques, namely tDCS, rTMS, and iTBS, on distinct motor domains (upper limb, lower limb, and vocal/swallowing functions) in chronic stroke patients, a population often underrepresented in previous reviews.

## Methods

We performed this systematic review to explore the existing evidence on the use of NIBS in chronic stroke patients to improve motor outcomes. The methodological approach was guided by the recommendations of Pollock and Berge [25]. This systematic review was performed following the recommendations of the Preferred Reporting Items for Systematic Reviews and Meta-Analyses (PRISMA) guidelines [26], with a registration number in the Prospective Register of Systematic Reviews CRD42023458370 (PROSPERO 2023). The data from this study were collected as part of a larger study, evaluating motor and cognitive outcomes following NIBS, and the data will be published separately for the two outcomes.

### PICO model

We used the PICO (Population, Intervention, Comparison, Outcome) model [27] to define the research question. Our research questions were as follows: “Is NIBS a feasible and safe tool in the neurorehabilitation of chronic stroke patients?” and “What are the optimal treatment approaches for stroke patients undergoing NIBS?”. To investigate these questions, we selected evidence with adults (>18 years) patients affected by chronic stroke (both ischaemic and haemorrhagic), as population; the intervention included all non-invasive neuromodulation techniques (e.g., TMS, rTMS, i-TBS, tDCS); the comparison included sham or placebo stimulation conducted in the control group, allowing for a comparative analysis of the effects of the active interventions; and outcomes included any motor improvements in the following functions: upper limb, lower limb (e.g., gait and balance), vocal and swallowing, shown by the patients and efficacy of treatment.

### Search strategy and information sources

A systematic search, according to PRISMA guidelines [26], was conducted for all peer-reviewed articles published from January 2010 through September 2023, using the following databases: PubMed, Embase, Cochrane Database of Systematic Reviews, PEDro, RehabData, and Web of Science. The following terms were used: (“chronic stroke and neuromodulation”, and “neuroplasticity”) AND (“transcranial non-invasive stimulation” OR “NIBS”) AND/OR (“Transcranial magnetic stimulation “ OR “TMS”) AND/OR (“Transcranial direct current stimulation” or “tDCS”).

### Eligibility criteria

The inclusion criteria were: (i) adult patients with chronic stroke; (ii) an applied approach to cognitive and motor rehabilitation; (iii) the English language; and (v) published in a peer-reviewed journal. We have excluded articles that describe theoretical models, including methodological approaches, algorithms, and basic technical descriptions, focusing instead on empirical and applied studies that present concrete and verifiable results. Additionally, we excluded: (i) animal studies; (ii) conference proceedings or reviews; and (iii) studies involving children; (iv) case reports and reviews. The list of articles was then refined for relevance, revised, and summarized, with the key topics identified from the summary based on the inclusion/exclusion criteria.

### Selection and data collection processes

Two independent reviewers (M.G.M. and M.B.) screened titles, abstracts, and full texts, applying inclusion and exclusion criteria under blinded conditions to reduce selection and publication bias. Reviewers were blinded to the names, affiliations, and journals of the authors. Discrepancies were discussed and resolved with a third reviewer (R.S.C.). Inter-rater agreement was calculated using the kappa statistic, with κ > 0.61 indicating substantial concordance. [21]

### Data items and data extraction

After the preliminary articles’ selection process, data from the included studies were extracted and organized into a summary table using Microsoft Excel (Version 2021). In accordance with PRISMA guidelines, rigorous procedures were employed for data extraction to ensure comprehensive coverage of relevant information. The extracted information included: assigned ID number, study title, year of publication or presentation and first author, study aims and design, study duration, recruitment method and setting, inclusion and exclusion criteria, presence of a control group, use of devices, informed consent, conflicts of interest and funding sources, type of intervention and control, number of participants, baseline characteristics, intervention setting, outcome measures and time-points for assessment, adverse events, results, and key conclusions. Once summarized, the data were uploaded into an online database (RYYAN) [28], where reviewers independently evaluated the relevance of each study based on titles, abstracts, and full texts. This meticulous approach ensured a thorough and reliable synthesis of the available evidence, enhancing the robustness of our review.

## Results

### Assessing quality of included studies – Risk of bias

The risk of bias in controlled studies was assessed through a revised Cochrane risk of bias (RoB 2) [29], which consists of five domains: i) bias arising from the randomization process, ii) bias due to deviations from intended intervention, iii) bias due to missing outcome data, iv) bias in the measurement of the outcome, v) bias in the selection of the reported result. The screening and assessment were independently performed by two reviewers, who were blinded to each other’s evaluations during this stage to minimize potential bias. Once the initial assessments were completed, the blinding was removed. In cases where discrepancies between the reviewers’ assessments arose, a third reviewer mediated the discussion to reach a consensus. As a result from our analysis, the majority of the included studies (45 out of 58) were rated with a low risk of bias, as they reported robust methodologies (see S1 for the RoB analysis of the included studies).

### Upper limb

The most prevalent risk of bias was in domain 1, related to randomization processes, in which only 16 [30–44] out of 26 studies were judged as having low risk. The remaining 10 studies were rated as having moderate [45,46] to serious [47] concerns regarding randomization procedures, primarily due to insufficient reporting of key methodological details, such as the method used to generate the random allocation sequence, the type of randomization, the allocation concealment mechanism, and whether allocation was blinded. One of these studies [47] was assessed with a serious concern, as no specific randomization procedure was described. Instead, participants were assigned to the experimental or control group in a seemingly random manner without the use of stratified randomization. This approach raises a potential risk of bias, particularly in terms of achieving balanced groups with respect to baseline functional severity. In contrast, the other 16 studies [30–44] with a low risk of bias reported information related to the type of procedure used and the accuracy of recruitment.

**Fig 1:**
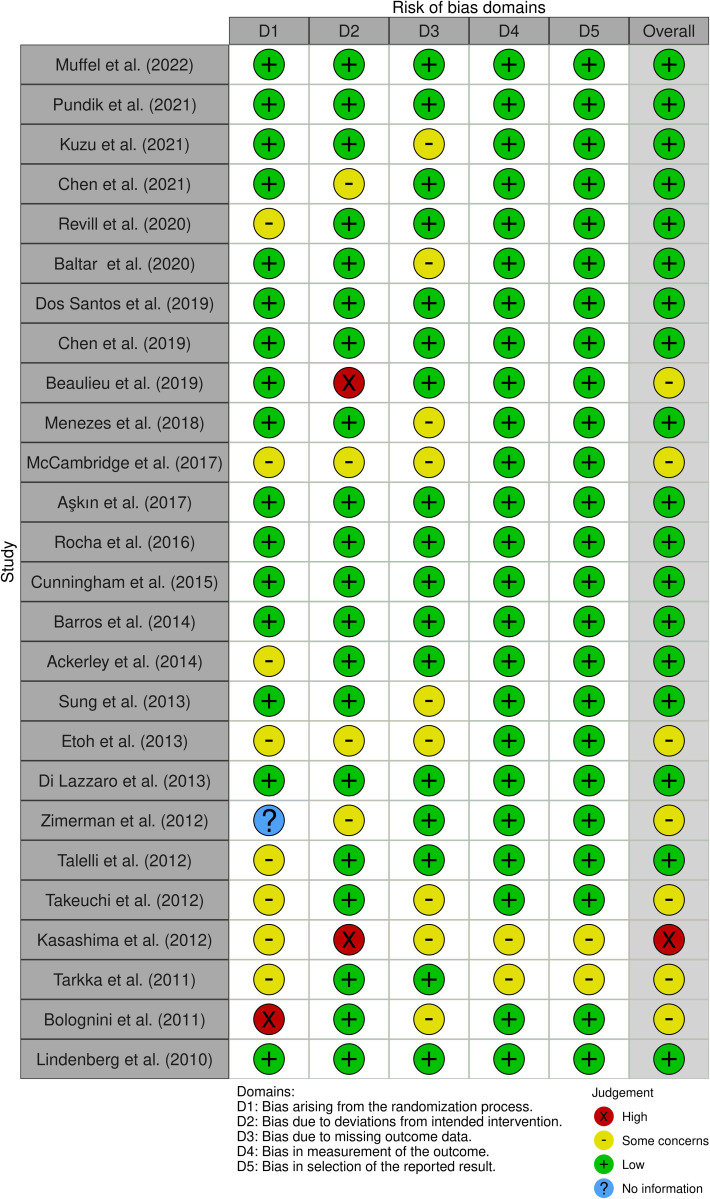
ROB2 Upper Limb.

Despite this, 6 studies [33,37,48–51] presented biases in domain 2, because they did not report blinding or did not specify who was blinded. In domain 3, 9 studies [32,34,42,47–49,51–53] were judged to have some concerns regarding bias control, particularly due to a lack of transparency in handling missing data. One recurrent limitation was the insufficient adherence to standardized reporting frameworks, such as the CONSORT statement, which recommends practices like intention-to-treat analysis and detailed reporting of attrition. These omissions can undermine the interpretability and reproducibility of results. In 9 out of the selected articles [32,34,42,47–49,51–53], the absence of structured reporting limited confidence in the handling of missing data, raising concerns regarding the potential risk of bias in domain 3. Furthermore, domains 4 and 5 were generally rated as low risk of bias, except for two studies [51,54], which raised some concerns due to the limited number and inconsistency of outcome measures relative to their stated objectives (Fig 1).

### Lower limb and walking functions

**Fig 2:**
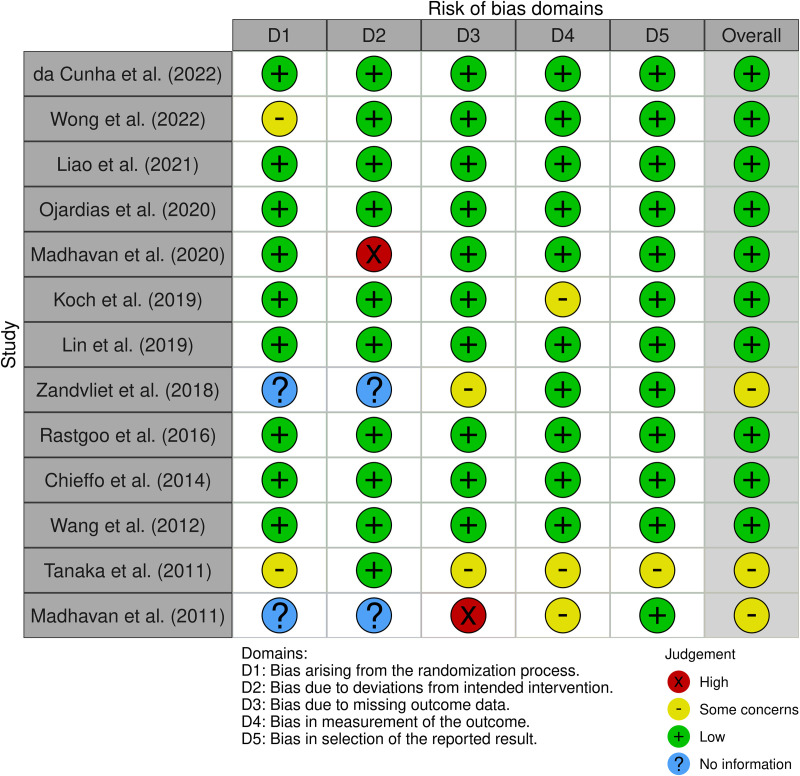
ROB2 Lower limb and walking functions.

The most prevalent risk of bias was in domain 2, because in two studies [55,56] it was not reported that blinding procedures were used, and in one study [56] there was no blinding. Furthermore, in domain 3, two studies [55,56] were judged to have some concerns regarding control of bias, and in one, the risk was judged to be high. Moreover, in domains 4 and 5, we consider the studies to have low bias, except for some concerns in three studies [56–58] for domain 4 and one [58] in domain 5, where outcome measures were few, and in some cases inconsistent with goals (Fig 2). For example, Tanaka et al. evaluated knee extension muscle force as the primary outcome and assessed hand-grip strength as a secondary measure that was quite inconsistent with the primary goal.

**Fig 3:**
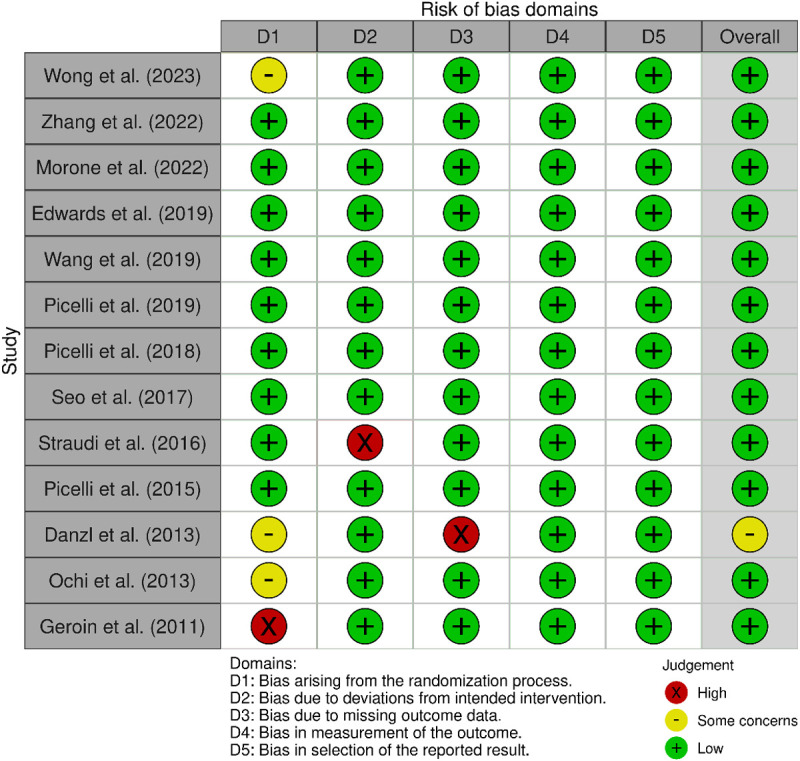
ROB2 of Robotic-assisted therapy combined with neuromodulation.

### Robotic-assisted therapy combined with neuromodulation

In Domain 1 (randomization process), 54 out of 58 studies were judged to be at low risk of bias. Three studies [59–61] raised some concerns due to the lack of description of the randomization procedure and, in one case [61], there was no information about allocation. One study [62] was rated as high risk due to the complete absence of details on randomization procedures and allocation methods. Regarding bias in Domain 2, Straudi et al., [63] did not specify the blinding procedures or clarify which individuals were blinded to the evaluation or treatment, raising concerns about potential performance and detection bias. In particular, Danzl et al. [60] did not implement specific strategies to address or mitigate potential bias related to missing outcome data.

A visual summary of risk of bias across all studies and domains is provided in Fig 3.

**Fig 4:**
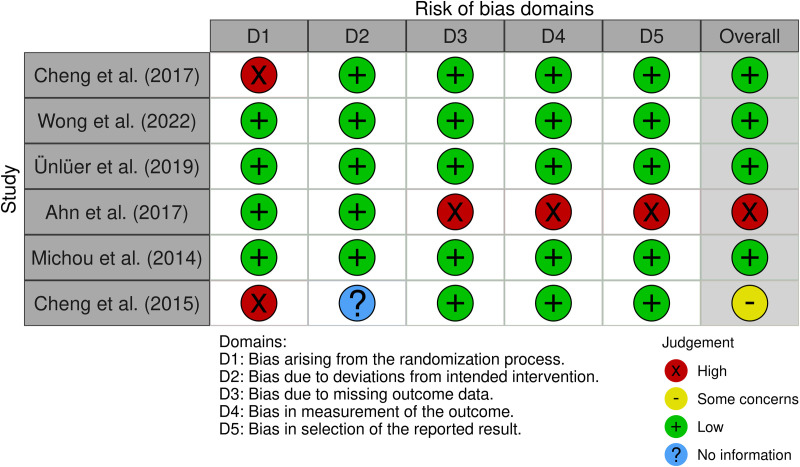
ROB2 of Vocal and swallowing functions.

### Vocal and swallowing functions

Most studies in this category were judged to be at low risk of bias across the assessed domains. However, one study [64] was rated as high risk due to the absence of any bias control measures, limited outcome measures, and poor alignment between the outcomes assessed and the study objectives. Additionally, in Domain 1 (randomization process), two studies [65,66] did not report any randomization procedures, raising concerns about selection bias (Fig 4). Overall, conducting a robust quality assessment for this functional category was challenging due to the limited number of included studies. While several studies demonstrated a low risk of bias, others exhibited moderate to serious methodological difficulties, particularly in randomization, masking, outcome selection, and bias control, which reduce their reliability and should be interpreted with caution.

**Fig 5:**
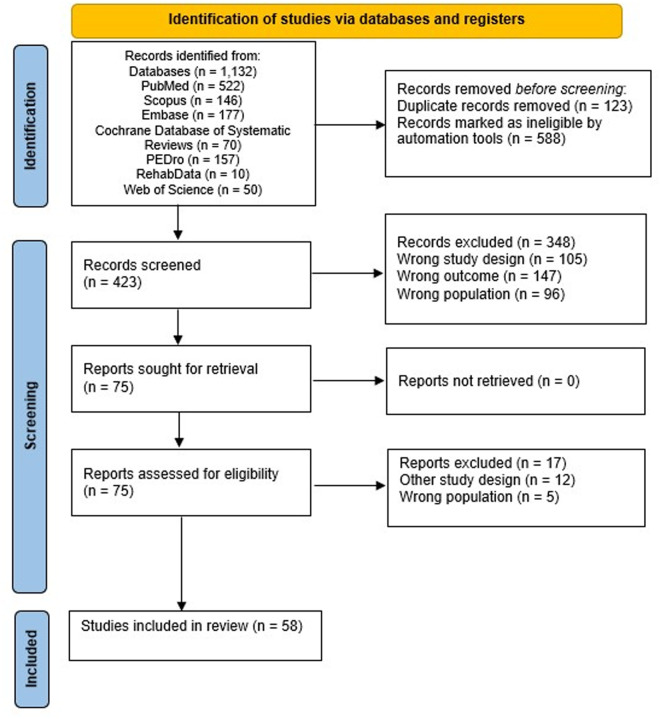
PRISMA flow chart.

### Synthesis of evidence

Electronic searches identified 1.132 papers. We assessed and included 75 studies, according to their pertinence and relevance to the topic. After reading the full text of the selected publication and applying the predefined inclusion criteria, we excluded 17 articles, and 58 articles were included in our qualitative analysis (see Fig 5) (see S2 and S3 for all excluded and included manuscripts). We divided our results in four sections as follows: (1) upper limb, (2) lower limb, (3) robotic assisted therapy combined with neuromodulation, and (4) vocal and swallowing functions.. The summary presented below reflects the relative distribution of studies included across functional domains: 26 studies [30–54,67] focused on upper limb rehabilitation, 13 studies [55,56,58,68–77] on Lower limb and walking functions rehabilitation, 13 [60–63,11,78–85] on Robotic-assisted therapy combined with neuromodulation, and 6 [59,64–66,86,87] on Vocal and swallowing functions. Therefore, the level of detail varies proportionally to the available evidence within each category, to ensure accuracy without over-interpreting limited data.

### Upper limb

Our search revealed 26 [30–54, 67] articles focusing on the use of NIBS, including i-TBS, rTMS, and tDCS for the rehabilitation treatment of upper limb functions in 684 chronic post-stroke patients. In particular, we found 25 RCTs [30,32,34–54, 67], 1 pilot study [33], and 1 randomised cross-over study [31]. The sample size ranged from 6 to 80 patients. In particular, the intervention was carried out through i-TBS, rTMS, and tDCS. In detail, 5 RCTs [32,33,43,46,67] investigated the effects of i-TBS in chronic stroke patients. Each study addressed the brain stimulation on the ipsilateral or contralateral motor cortex according to inhibitory or excitatory stimulation, after conventional physiotherapy. In addition, Kuzu et al. [32] examined the effects of i-TBS and low-frequency rTMS combined with physiotherapy. The authors [32] suggested that both techniques were effective in improving upper limb functions, but benefits are limited for spasticity. Both the real rTMS and real TBS groups showed significant improvements in total upper extremity Fugl-Meyer (FM) scores both post-treatment (p = 0.017 for both groups) and at the 4-week follow-up (p = 0.18 for rTMS and p = 0.018 for cTBS). The real rTMS group exhibited significant improvements in spasticity scores, measured with Modified Ashworth Scale (MAS), for the elbow flexor, pronator, wrist flexor, and finger flexor muscle groups post-treatment (p = 0.025, p = 0.025, p = 0.039, p = 0.038, respectively) and at the 4-week follow-up (p = 0.025, p = 0.025, p = 0.039, p = 0.038, respectively). The real TBS group showed significant improvements in MAS scores for the elbow flexor and wrist flexor muscle groups post-treatment (p = 0.025, p = 0.034, respectively), and for the wrist flexor at the 4-week follow-up (p = 0.024).

On the other hand, 9 studies [31,38,39,41,45,46,53,54] (8 RCTs and one randomized cross-over study) investigated the effects of rTMS on the paretic upper limb. In 5 studies [31,35,38,39,41] out of 9 addressed rTMS on the contra lesional/unaffected primary motor cortex, and in 2 of these [35,39] authors associated conventional physiotherapy with rTMS. One study Aşkın A, Tosun A, Demirdal ÜS. Effects of low-frequency repetitive transcranial magnetic stimulation on upper extremity motor recovery and functional outcomes in chronic stroke patients: A randomized controlled trial. Somatosens Mot Res. 2017;34(2):102–107. https://doi.org/10.1080/08990220.2017.1316254 administered low-frequency rTMS (LF-rTMS) stimulating contra lesional primary motor cortex, in association with conventional physiotherapy and reach-to-grasp training. Revill et al. [45] used a particular rTMS technique called Hebbian, which has been used to induce long term potentiation in postsynaptic pyramidal tract neurons. Interestingly, these authors found that significant correlations occurred between improvements in hand function and changes in motor cortex (M1) activity for both hemispheres (p < 0.05). Participants who showed greater improvement in hand function also had greater increases in task-related M1 activity (p < 0.05). These findings were particularly strong in the treatment group with the Hebbian-based training compared to the sham therapy, suggesting that this approach led to both functional and neural improvements. In addition, Takeuchi et al. [53] combined LF-rTMS with anodal tDCS to prevent deterioration of coordination of bimanual movements.

The remaining twelve studies investigated the effects of tDCS on upper limb functions in chronic stroke. Half of them administered bi- or mono-hemispheric anodal and cathodal tDCS on the primary motor cortex. The other two studies combined tDCS with physiotherapy [45] and with a resistance training program [37] to improve upper limb functions.

Rocha et al. [39] and Bolognini et al. [47] administered cathodal tDCS in addition to constraint-induced movement therapy (CIMT).

Moreover, Chen et al. [33] suggested that tDCS-iTBS combined physical therapy can be a promising approach to enhance therapeutic benefits of upper limb treatment. In particular, these authors, found significant improvements in functional hand motor skills, measured with Jebsen-Taylor hand function test (JTT) (*p* = 0. 016), as well as in upper limb coordination, measured with finger-to-nose test (FNT) (*p* = 0. 037). Also, Menezes et al. [52] applied tDCS in combination with repetitive peripheral nerve sensory stimulation (RPSS) could enhance the effects of functional electric stimulation on active range of movement of paretic wrist, (see Table 1). They found that the real NIBS group had greater improvements in the JTT (p = 0. 016) and FNT (p = 0. 037) scores than the sham NIBS group.

**Table 1:** Description of studies regarding the use of NIBS on upper limb outcomes.

Ref. n°	Study design	Sample size	Intervention		Type of NIBS	Outcomes	Major findings	PEDro score
			EG	CG				
25	RCT	34	tDCS was applied concurrently to task execution in the KINARM Robotic environment	sham-controlled	tDCS	KINARM exoskeleton lab	tDCS impacts post-stroke sensorimotor functions	10
26	RCT	60	3 rTMS treatments targeted contralesional S1: Sham, 5 Hz and 1 Hz. The rTMS was delivered simultaneously with peripheral sensory electrical stimulation and vibration of the affected hand.	–	rTMS	Disk-Criminator disks Proprioception evaluation TSWM BTM SEP	Short-term application of facilitatory high frequency rTMS (5 Hz) with peripheral somatosensory stimulation may promote somatosensory function	10
27	RCT	30 EG1:7EG2:7 CG: 6	Ten sessions of real rTMS or real cTBS combined with PT	PT	rTMS	MAS FMA-UE FIM MAL-28 BUE HMRS	rTMS has beneficial effects in motor functional recovery and daily living activities both at post-treatment and follow up at 4 weeks	8
28	RCT	24 EG:12 CG:12	18 treatment sessions of 1 h of a conventional rehabilitation program (3 days a week for 6 weeks), where a 20-min real NIBS intervention was simultaneously applied during conventional rehabilitation.	18 treatment sessions of 1 h of a conventional rehabilitation program (3 days a week for 6 weeks), where a 20-min sham NIBS intervention	tDCS iTBS output	FMA-UE JTT FNT	Patients undergoing combined ipsilesional tDCS-iTBS stimulation with conventional rehabilitation showed greater impacts than patients undergoing sham stimulation-conventional rehabilitation	8
29	RCT	20 EG:10 CG:10	5 days of hand motor training that was combined with either Hebbian-type (trainingHebb) of the lesioned M1	5 days of hand motor training that was combined with sham stimulation of the lesioned M1	rTMS Hebbian-type stimulation	JTT MAL-28 Brain Imaging	motor training combined with Hebbian-type stimulation increases functional outcomes and allows maintenance over time.	9
30	RCT	80	ten to fifteen sessions of tDCS plus physical therapy	–	tDCS	UE-FM	UE-FM evaluation may be predictive for clinical motor improvement induced by tDCS	10
31	RCT	20 EG 10 CG:10	1 Hz rTMS on the unaffected hemisphere and PT, for ten sessions.	Sham stimulation and PT, for ten sessions.	rTMS	MAS MSO	1-htz rTMS and PT increased the unaffected hemisphere excitability, decreased spinal excitability, and reduced ULS.	10
32	RCT	22 EG:11 CG:11	1 session per day for 10 days of either iTBS over the ipsilesional primary motor cortex and conventional neurorehabilitation.	1 session per day for 10 days of sham stimulation over the ipsilesional primary motor cortex and conventional neurorehabilitation	iTBS	MAS FMA-UE ARAT BBT MAL	iTBS induced greater gains in spasticity decrease and UL function improvement, especially in fine motor function, than sham iTBS	10
33	RCT	14 EG: 7 CG : 7	real tDCS + resistance training	sham tDCS + resistance training	tDCS	FMA-UE BBT WMFT grip strength MAS MAL	Repeated sessions of bi-hemispheric tDCS combined with resistance training are safe and tolerable	7
34	RCT CROSSOVER	20	active RPSS+active tDCS active RPSS+sham tDCS sham RPSS+active tDCS sham RPSS+sham tDCS		tDCS	NIHSS MRS MMSE Oldfield inventory MAS	Single sessions of PSS + tDCS, tDCS alone or RPSS alone did not improve training effects	9
35	RCT	10 EG:5 CG:5	anodal, cathodal, in separate sessions with the ‘target’ electrode positioned over the contralesional M1 (C) and ‘reference’ electrode on the ipsilesional (I) forehead	sham tDCS	tDCS	FMA UE ARAT MAS	Anodal tDCS increased contralesional corticomotor excitability Cathodal tDCS did not affect corticomotor excitability	6
36	RCT	40 EG:20 CG:20	rTMS (1 Hz, 1200 pulses) to the unaffected hemisphere for 20 min. Each patient received a total of 10 sessions in 2 weeks (5 days/week) before PT sessions.	Sham rTMS	rTMS	BRS FMA-UE BBT MAS FIM MMSE FAS	TMS seems to be a promising treatment for motor, functional, and cognitive deficits in chronic stroke.	9
37	RCT	21 EG1:7EG2:7 CG:7	(EG1) anodal (EG2) cathodal (CG) sham tDCS Each combined with mCIMT		tDCS	FMA MAL handgrip strength	Combining mCIMT with brain stimulation increases clinical gains in stroke rehabilitation. However, anodal tDCS appears to have a greater impact than cathodal tDCS	10
38	RCT	12 EG: 6 CG: 6	tDCS to the ipsilesional higher motor areas in rehabilitation	Sham tDCS	tDCS	FMA-UE NHPT MAL	Stimulation of higher motor areas can recruit the contralesional hemisphere into an adaptive role in cases of major ipsilesional injury	10
39	RCT	20 EG:10 CG:10	rTMS to the primary motor cortex of the unaffected side (1500 pulses; 1 Hz; 90% of resting motor threshold for the first dorsal interosseous muscle) in 10 sessions, and PT	sham stimulation and PT	rTMS	MAS FMA-UE SSQOL	rTMS associated with PT can be beneficial in reducing poststroke spasticity	10
40	RCT CROSSOVER	12	ipsilesional M1 intermittent TBS (iTBSiM1), contralesional M1 continuous TBS (cTBScM1) or sham TBS		iTBS	grip-lift performance MEPs	Primary motor cortex iTBS not only modulates M1 corticospinal excitability but also increases M1 receptiveness to sensory input	9
41	RCT	54	20 daily sessions of (group1) 1 Hz rTMS over the contralesional primary motor cortex (M1) and iTBS over the contralesional primary motor cortex; (group2) contralesional sham stimulation and ipsilesional real iTBS; (group3) 1 Hz contralesional real rTMS and ipsilesional sham stimulation; (group4) bilateral sham control procedures		rTMS iTBS	FMA-UE WMFT MRCS RT FT	Stimulation can be delivered over an extended interval of time safely and results favorably in facilitating motor performance and improving interhemispheric imbalance.	9
42	RCT	18 EG:9 CG:9	motor rTMS sessions for two weeks, followed by sham rTMS sessions for two weeks	sham rTMS sessions for two weeks, followed by motor rTMS sessions for two weeks	rTMS	FMA-UE ARAT MAS STEF	Multiple sessions of 1-Hz rTMS facilitated the effects of repetitive facilitation exercises in improving motor function of the affected upper limb but did not change spasticity.	7
43	RCT	12 EG:6 CG:6	Active rTMS used cTBS- 3 pulses at 50 Hz, repeated every 200 ms for a total of 600 pulses.	sham stimulation	rTMS cTBS	ARAT JTT NHPT	Ipsilesional inhibitory TBS is safe and can be used in a study to increase the gain from a late rehabilitation program in chronic stroke patients.	10
44	RCT Crossover	12	cathodal tDCS were applied to the contralesional motor cortex	sham intervention applied to the contralesional motor cortex	tDCS	MMSE MRC FMA-UE ASS	tDCS is a promising tool to improve not only motor behavior, but also procedural learning.	7
45	RCT	41	EG1: excitatory TBS to the ipsilesional hemisphere; EG2: inhibitory TBS to the contralesional hemisphere. CG: sham TBS. TBS was followed by physical therapy for 10 working days		TBS	NHPT JTT grip and pinch-grip dynamometry	Cortical stimulation did not augment the gains from a late rehabilitation program	9
46	RCT	27	EG1: 1 Hz rTMS over the unaffected hemisphere EG2: anodal tDCS over the affected hemisphere EG3: a combination of rTMS and tDCS		rTMS tDCS	MEP TCI	The short-term decline was prevented by combined with low frequency rTMS to the unaffected hemisphere and anodal tDCS to the affected hemisphere.	8
47	RCT	6	anodal tDCS (10 min, 1 mA) over the affected primary motor cortex in a random order	sham stimulation over the affected primary motor cortex	tDCS	ERD EEG	anodal tDCS may increase mu ERD in hemiparetic stroke patients.	5
48	RCT	20	functional electrical therapy and upper limb treatment (twice daily sessions) for two weeks	conventional physiotherapy and upper limb treatment (twice daily sessions) for two weeks	TMS	WMFT MEP	functional exercise augmented with individualized electrical therapy of the paretic upper limb may enhance neuroplasticity	7
49	RCT	14	tDCS (cathodal stimulation of the unaffected motor cortex and anodal stimulation of the affected motor cortex), combined with CIMT	Sham stimulation	tDCS CIMT	JTT HS MAL FMA-UE BIS BDI TMS	Bihemispheric tDCS can achieve greater functional recovery, modulate local excitability, and remove the imbalance in transcallosal inhibition. CIMT can only modulate local excitability.	6
50	RCT	20 EG:10 CG:10	5 consecutive sessions of bihemispheric transcranial direct current stimulation (tDCS) (anodal tDCS and cathodal tDCS with simultaneous physical/occupational therapy	sham stimulation with simultaneous physical/occupational therapy.	tDCS	WMFT FMA-UE	The combination of bihemispheric tDCS and peripheral sensorimotor activities improved motor functions	11

* Legend: Action Research Arm Test (ARAT), Ashworth Spasticity Scale (ASS), Barthel Index Score (BIS), Beck Depression Inventory (BDI), Bimanual movement and transcallosal inhibition (TCI), Box and Block test (BBT), Biothesiometer (BTM); Brunnstrom upper extremity (BUE); constraint-induced movement therapy (CIMT), continuous Theta Burst Stimulation (cTBS); Event-related Desynchronization (ERD), Finger-to-Nose Test (FNT), Functional Independence Measure (FIM), Fugl-Meyer Assessment-Upper Extremity (FMA-UE), Functional Ambulation Scale (FAS), Hand Motor Recovery Stage (HMRS), Handgrip Strength (HS), Index Finger Tapping Task (FT), Jebsen-Taylor Hand Function Test (JTT), Mini-Mental State Examination (MMSE), Motor Activity Log-28 (MAL-28), Modified Ashworth Scale (MAS), Modified Constraint-induced Movement Therapy (mCIMT), Modified Rankin Scale (MRS), Motor evoked potentials (MEP), Medical Research Council Scale (MRCS); Nine-hole Peg Test (NHPT), Output Intensity Of The Magnetic Stimulator (MSO), Physical Therapy (PT), Simple Reaction Time Task (RT), Simple Test For Evaluating Hand Function (STEF), Stroke-specific Quality-of-life Scale (SSQOL), Somatosensory Evoked Potentials (SEP), Tactile Semmes-Weinstein Monofilaments (TSWM), Wolf Motor Function Test (WMFT).

### Lower limb function

In addition to upper limb rehabilitation, another key area investigated was lower limb function. We found 13 articles [55,56,58,68–77] dealing with the use of NIBS to promote balance and gait recovery in 267 chronic post-stroke patients. Specifically, our research found 8 RCTs [55,56,69–71,73,75,77], 3 clinical trials [58,68,72], 2 pilot studies [74,76]. The sample size of the selected RCTs ranges from 10 to 48 patients. The studies included investigated different types of NIBS, including i-TBS, rTMS and tDCS (see Table 2).

**Table 2:** Description of studies regarding the use of NIBS on gait and balance outcomes.

Ref. n°	Study design	Sample size	Intervention		Type of NIBS	Outcomes	Major findings	PEDro score
			EG	CG				
51	Clinical trial	32	10 concurrent tDCS and FDS gait training, 5 times per week for 2 weeks.	sham stimulation	tDCS	TUG	Bicephalic tDCS does not add relevant benefits to FDS and gait training	10
52	RCT	48	anodal tDCS group (*n *= 12), bilateral tDCS group (*n *= 12), cathodal tDCS group (*n *= 12)	sham tDCS group (*n *= 12)	tDCS	CDT MDT Cadence step time step length	one-session of tDCS increased contralesional corticomotor inhibition and improved dual task gait performance	7
53	RCT	30	PT 5 times per week for 2 weeks, and cerebellar iTBS	PT 5 times per week for 2 weeks, and sham iTBS	i-TBS	BBS, TIS FMA-LE BI	Cerebellar iTBS with PT promotes balance and motor recovery	10
54	RCT	18	single session of anodal stimulation (2 mA, 20 min) over M1-LL (a-tDCS condition)	pseudostimulation session (SHAM condition)	tDCS	Wade test 6MWT GAITRite, posturography	a single session of anodal tDCS of M1-LL can have a positive effect in chronic hemiplegic patients.	10
55	Clinical trial	9	Anodal tDCS over the lesioned LL M1, anodal tDCS over the non-lesioned LLM1	sham tDCS over the lesioned lower limb M1	i-TBS	MMSE Electromiography MEP	NIBS applied to the lesioned motor cortex enhances voluntary control of the paretic ankle	7
56	RCT	36	iTBS applied over the cerebellar hemisphere ipsilateral to the affected body side	sham iTBS	i-TBS	FMA-LE, BBS and BI	Patients treated with iTBS, but not with sham iTBS, showed improvement of gait and balance functions	8
57	Pilot study	20	10 sessions of iTBS group (5-week)	10 sessions sham group over a 5-week period	iTBS	MEP NIHSS mRS BRS BBS TUG FMA-LE	There was no powerful evidence to support the effectiveness of iTBS group better than sham control group.	10
58	RCT	15	Anodal stimulation on the contra-lesional cerebellar hemisphere, ipsi-lesional cerebellar hemisphere, for 20 min with 1.5 mA in three sessions	sham stimulation for 20 min	tDCS	BBS FRT TUG FRT FES EmNSA-LE	Contra-lesional cerebellar tDCS shows promise for improving standing balance performance.	4
59	RCT	20	5 consecutive daily sessions of active rTMS to the unaffected lower extremity motor area (1000 pulses; 1 Hz; 90% of the tibialis anterior motor threshold).	five consecutive daily sessions of sham rTMS	rTMS	MMAS H-reflex FMA-LE TUG	Low frequency rTMS over the LE motor area can improve clinical measures of muscle spasticity and motor function.	10
60	Pilot study	10	11 sessions of Real rTMS in a random sequence, over 3 weeks and separated by a 4-week washout period	11 sessions of sham rTMS	rTMS	FMA-LE 10MWT 6MWT	3 weeks of high-frequency deep rTMS could induce long-term improvements in LL functions	11
61	RCT	24	Repetitive TMS (1-Hz frequency) over the leg area of the motor cortex of the unaffected hemisphere for 10 minutes	sham TMS	rTMS	MEP FMA-LE gait performance	rTMS enhances the effect of task-oriented training, increasing gait spatial symmetry and corticomotor excitability symmetry.	10
62	Clinical trial	8	anodal tDCS of the LL motor cortex representation in the affected hemisphere	sham tDCS of the LL motor cortex	tDCS	VAS MF-LE	Anodal tDCS transiently enhanced knee extensor strength.	5
63	RCT	9	Facilitatory tDCS in a random order over LL primary motor cortex of the lesioned hemisphere or the non-lesioned hemisphere	sham stimulation over the lesioned hemisphere	tDCS	FMA-LE MMSE EMG	tDCS applied to the lesioned motor cortex enhances voluntary control of the paretic ankle.	3

*Legend: Berg Balance Scale (BBS), Brunnstrom Stage (BRS), Cognitive Dual Task walking (CDT), Electromyographic (EMG), Fall Efficacy Scale (FES), Fugl-Meyer Assessment Limb Extremity (FMA-LE), Functional Reach Task (FRT), Footdrop Stimulator (FDS), Lower Limb (LL), maximum force of the lower extremities (MF-LE), modified Rankin Scale (mRS), Modified Ashworth Scale (MMAS), Motor Evoked Potentials (MEP), Motor Dual-task Walking (MDT), National Institute of Health Stroke Scale (NIHSS), Nottingham Sensory Assessment of the Lower Extremity (EmNSA-LE), Physiotherapy (PT), Timed “Up & Go” test (TUG), Visual analogue scale (VAS), 6-minute walk test (6MWT), 10-m walk test (10MWT).

Firstly, the i-TBS was used in four studies [70,72–74] (two RCTs and two pilot studies) to promote gait and balance recovery stimulating cerebellar [70,73] and bilateral cerebral motor cortex [72]. Each study coupled the brain stimulation with conventional physiotherapy. Physiotherapy sessions were arranged immediately after the i-TBS stimulation. Additionally, patients enrolled in the studies [70,72,73] presented moderate motor deficits and were randomly assigned to i-TBS or sham groups.

The rTMS and TMS were performed in three studies [75–77] (2 RCTs and 1 pilot study) to improve lower limb functions, walking ability and spasticity. Specifically, only Chieffo et al. [76] administered rTMS using the H-coil which is designed to reach deep brain regions. Study comparisons were made with sham controls in all three studies and Wang et al. [77] performed a task-oriented gait training after brain stimulation.

Lastly, tDCS was used in 7 studies [55,56,58,68,69,71,72] (5 RCTs and 2 controlled clinical trials) to improve walking, balance abilities and muscle strength in lower limb. Combined approach was used by da Cunha et al. [68], who administered tDCS, footdrop stimulation and gait training simultaneously, while Madhavan et al administered tDCS with treadmill training. Specifically, da Cunha et al., [68] found statistically significant time effects were found in clinical tests to evaluate gait and balance functions, such as the Timed-Up-Go (TUG) (p < 0.001), while for spasticity, a main effect of time was seen for plantar flexors and knee extensors on the MAS (P < .05), with reductions in resistance in both groups over time. In addition, walking distance improved significantly over time (p = 0.001) in both groups, with an average increase of 76 meters, indicating clinical relevance. Moreover, Madhavan et al., [72] found that he tDCS plus ankle motor tracking group showed significantly greater increases in corticomotor excitability, compared to other groups (e.g., high-intensity speed-based treadmill training) (*p* = 0.02), suggesting enhanced neuroplastic effects. Zandvliet et al. [55] stimulated cerebellar cortex using tDCS during medio-lateral postural tracking task on force platform to improve balance performance in post-stroke patients. In particular, they found that after the tDCS stimulation, several centre of pressure (CoP) measurements were significant improved, including CoP amplitude (*p* = 0.02), CoP variability (*p* = 0.01), CoP range and velocity (*p* = 0.01, p = 0.02). Tanaka et al. [58] and Ojardias et al. [71], used anodal tDCS stimulation to improve respectively knee extension strength (*p* < 0.01) and walking abilities tested with 6-minutes walking test (6MWT) (p = 0.038), compared to the sham therapy (see Table 2).

### Robotic assisted therapy combined with neuromodulation

Beyond domain-specific functional targets, some studies have explored the synergistic effects of combining NIBS with other rehabilitation modalities. In particular, we identified 13 RCTs [60–63,11, 78–85] dealing with robot assisted therapy (RAT) combined with neuromodulation in 397 chronic post-stroke patients. In 5 RCTs [61,63,78–80] RAT was focused on the treatment of upper limb, while in the remaining 8 [60,62,11,81–85] RCTs RAT was focused on gait training.

In the 5 RCTs [78,79], sample size ranged between 18 and 82 chronic post-stroke patients. The intervention regarded the use of RAT for upper limbs in combination with tDCS to improve manual dexterity. Specifically, in 3 studies [63,79,80] compared tDCS with sham stimulations plus RAT, except for Ochi et al. [61], which compared anodal and cathodal tDCS using a cross-over study design. The RAT was carried out through different robotic devices including exoskeleton like Armeo Power II [79], and end-effectors such as MIT Manus [80], REO therapy system [63] and Bi-Manu-Track robotic arm trainer (Reha-Stim, Berlin) [61]. In contrast, Zhang et al. [78] used two types of robotic devices such as one exoskeleton (HandRehab) and one end-effector (Armodus) combined with i-TBS to improve upper limb functioning. Specifically, they found a significant time-by-group interaction in FM scale scores (*p* = 0.011), with both priming and nonpriming iTBS outperforming sham stimulation. In patients with higher upper limb function, priming iTBS led to significantly greater FM improvements than both nonpriming iTBS (*p* = 0.025) and sham (*p *= 0.029).

The remaining 8 RCTs [60,62,11,81–85] were focused on the administration of RAT for gait function in chronic post-stroke patients. In details, sample size ranged between 8 and 45 chronic stroke patients. Interventions were carried out through tDCS in seven studies [60–63,79–81,11, 83–85] and rTMS in one study [82] in addition to robotic devices which included exoskeletons like Lokomat [60], Walbot_S [84], and end-effectors such as GE-O System [83,11,85] and Gait Trainer [62], except for Wong which used treadmill device (see Table 3).

**Table 3:** Description of studies regarding combined approach through NIBS and robotic-assisted training (RAT).

Ref. n°	Sample size	Intervention		Type of NIBS	Outcomes	Major findings	PEDro score
		EG	CG				
64	42	10 sessions of priming iTBS, nonpriming iTBS, or sham stimulation to the ipsilesional motor cortex, immediately before RobAT		iTBS	ARAT FMA-UE MMV	Priming and nonpriming iTBS are superior to sham stimulation in enhancing treatment gains from RobAT	10
65	66	dual tDCS immediately before RobAT (10 sessions, 2 weeks).	sham tDCS	tDCS	MEP FMA-UE	Adjunction of dual tDCS to RobAT did not further enhance recovery in the treated patients	10
66	82	tDCS (M1-SO montage, anode ipsilesional, 5 × 7 cm electrodes, 2 mA, 20 mins), prior RobAT	sham tDCS	tDCS	TMS FMA-UE	conventional tDCS does not confer further advantage to robotic training	10
67	23	RobAT + real tDCS 10 sessions (5 sessions a week) over two weeks.	RobAT + sham-tDCS	tDCS	BBT FMA-UE MAL	The additional use of bilateral tDCS to RAT seems to have a significant beneficial effect	10
68	18	anodal tDCS on the affected hemisphere and RobAT	cathodal tDCS on the unaffected hemisphere and RobAT	tDCS	FMA-UE MAS MAL	ombined therapy could achieve limited effects in the hemiplegic arm	8
69	45	EG1: a bilateral tDCS and treadmill training EG2: cathodal tDCS and treadmill training CG: a sham tDCS and treadmill training 50 min per session (20 min tDCS + 30 min treadmill) 3 sessions per week for 4 weeks.		tDCS	CDTW MDTW MEP FMA-UE	TDCS and treadmill training is an effective intervention for improving cognitive dual task walking and modulating contralesional cortical activity	8
70	14 EG:8 CG:6	5 Hz rTMS prior to treadmill training three times per week for 3 weeks	sham rTMS before treadmill training	rTMS	GAITRite system MEP FMA	rTMS over the leg motor cortex in the affected hemisphere enhanced the effects of subsequent treadmill training on gait speed and spatial asymmetry	10
71	40	on-line cathodal tDCS over the contralesional cerebellar hemisphere + cathodal transcutaneous spinal direct current stimulation.	on-line anodal tDCS over the damaged cerebral hemisphere + cathodal transcutaneous spinal direct current stimulation	tDCS	6MWT	cathodal tDCS over the contralesional cerebellar hemisphere in combination with cathodal transcutaneous spinal direct current stimulation may lead to similar effects on robAT	9
72	20	on-line cathodal tDCS over the contralesional cerebellar hemisphere + cathodal transcutaneous spinal direct current stimulation.	on-line anodal tDCS over the damaged cerebral hemisphere + cathodal transcutaneous spinal direct current stimulation	tDCS	6MWT	cathodal tDCS over the contralesional cerebellar hemisphere in combination with cathodal transcutaneous spinal direct current stimulation might be useful to boost the effects of RobAT	11
73	21	45 min after allocated tDCS on the leg motor cortex in the impaired hemisphere for 20 min every weekday for 2 weeks	sham tDCS	tDCS	6MWT 10MWT BBS FMA-LE MRCS MEP	anodal tDCS on the leg motor cortex in the impaired hemisphere may facilitate the effect of RobAT on functional ambulation	11
74	30	20-minute RobAT, five days a week, for 2 weeks combined with EG1: anodal tDCS + sham tsDCS CG: sham tDCS + cathodal tDCS EG2: tDCS + cathodal tDCS		tDCS	6MWT	anodal tDCS combined with cathodal tsDCS may be useful to improve the effects of robotic gait training	11
75	8	active tDCS for 12 sessions	sham tDCS for 12 sessions	tDCS	10MWT BBS TUG FAC SIS-16	tDCS has the potential to enhance the effectiveness of gait training	7
76	30	50-minute treatment sessions, five days a week, for two weeks. EG1: RobAT and tDCS EG2: RobAT and sham tDCS CG: walking exercises.		tDCS	6MWT 10MWT	tDCS had no additional effect on RobAT in patients with chronic stroke	8

*Legend: Berg Balance Scale (BBS), Cognitive (CDTW) and motor DTW (MDTW) Dual-task walking, Action Research Arm Test (ARAT), Band Block Test (BBT), Functional Ambulation Category (FAC), Fugl-Meyer Assessment-Upper/Lower Extremity (FMA-U/L E), Mean Movement Velocity (MMV), Medical Research Council Scale (MRCS), Modified Ashworth Scale (MAS), Motor Evoked Potentials (MEP), Motor Activity Log (MAL), Robot-assisted Training (RobAT), Stroke Impact Scale 16 (SIS-16), 6-minute walk test (6MWT), 10-m walk test (10MWT).

### Vocal and swallowing functions

In addition to limb-related motor functions, a smaller subset of studies investigated the use of NIBS for orofacial rehabilitation. We found 6 articles [59,64–66,86,87] focusing on the use of NIBS to promote swallowing skills, speech-related and vocal functions among post-stroke dysarthric patients. Specifically, our search revealed 5 RCTs [59,64–66,86] and 1 pilot study [87]. The sample size of the selected RCTs ranges from 4 to 26 patients. The studies included two types of NIBS (rTMS and tDCS).

rTMS was performed in four studies [65,66,86,87] (3 RCTs and one pilot study) to improve swallowing functions and dysphagia. Five studies used comparisons with sham controls [59,64–66,86]. Only Michou et al. [87] performed single pulse rTMS over the pharyngeal motor cortex and an assessment of pharyngeal electromyographic responses. Furthermore, they divided patients into three types of stimulation: electrical pharyngeal impaction (PES), paired associative stimulation (PAS), or rTMS. Instead, Chen et al., in an RCT and another pilot study [65,66], performed high frequency rTMS at 5 Hz applied to the tongue region of the motor cortex. Finally, only Ünlüer et al. [86] performed conventional dysphagia rehabilitation in both groups (sham and experimental), and then the experimental group also received 1 Hz rTMS in the unaffected hemisphere.

Finally, tDCS was used in 2 RCTs studies [59,64] to improve physiological, vocal, and swallowing function. Both studies used tDCS over the pharyngeal motor cortex and used experimental and sham groups. Only Ahn et al. [64] used conventional swallowing therapy in combination with tDCS (see Table 4).

**Table 4:** Description of the studies regarding the use of NIBS on vocal and swallowing outcomes.

Ref. n°	Study design	Sample size	Intervention		Type of NIBS	Outcomes	Major findings	PEDro score
			EG	CG				
77	RCT	15 EG:11 CG:4	3,000 pulses of 5 Hz rTMS on the tongue area of the motor cortex for 10 days over a period of 2 weeks.	Sham rTMS on the tongue area of the motor cortex for 10 days over a period of 2 weeks.	rTMS	MEP VFSS SWAL-QOL MTSM	5 Hz rTMS applied over the tongue area of the motor cortex is not effective for improving swallowing function	9
78	RCT	9	2mA of anodal tDCS over the left inferior primary motor cortex for 15 minutes	30s of stimulation under the same settings.	tDCS	HKCAT EMA	Anodal tDCS over the primary motor cortex has beneficial effects on speech production, and tDCS combined with speech therapy may promote recovery from dysarthria.	11
79	RCT	28	conventional dysphagia rehabilitation 3 days a week for 4 weeks, and 1 Hz rTMS to unaffected hemisphere in the final week.	conventional dysphagia rehabilitation 3 days a week for 4 weeks.	rTMS	NIHSS T-BI mRS VFSS NS SWAL-QOL SAFE PAS	rTMS did not enhance the swallowing function when compared conventional dysphagia rehabilitation.	11
80	RCT	26	bihemispheric anodal tDCS group anodal electrodes were attached bilaterally to the pharyngeal motor cortices, and cathodal electrodes were attached to both supraorbital regions	sham group	tDCS	DOSS	The bihemispheric anodal tDCS with conventional dysphagia therapy had additional helpful effects on the improvement in swallowing function in chronic stroke patients.	6
81	RCT	4 EG: 2 CG: 2	3,000 pulses of 5 Hz rTMS on the tongue area of the motor cortex for 10 days over a period of 2 weeks.	Sham group	rTMS		The study showed that excitatory rTMS applied over the tongue motor cortex is a feasible approach in individuals with chronic post-stroke dysphagia	4
82	Pilot study	18	single-pulse transcranial magnetic stimulation (TMS) over the pharyngeal motor cortex	Sham neurostimulation	rTMS	OTT, PRT, PTT, AC, UES	Authors showed that a single application of either PES or PAS increases cortical excitability and is associated with reductions in aspiration, while 5 Hz rTMS was less effective.	10

*Legend: Electromagnetic Articulography (EMA), Hong Kong Cantonese Articulation Test (HKCAT), Maximum Tongue Strength Measurement (MTSM), modifed Rankin Scale (mRS). Motor Evoked Potentials (MEP), National Institutes of Health Stroke Scale (NIHSS), Nutritional Status (NS), Penetration–Aspiration Scale (PAS), Swallowing Ability and Function Evaluation (SAFE), Swallowing Quality of Life questionnaire (SWAL-QOL), Turkish Barthel Index (T-BI), Videofluroscopic Swallowing Study (VFSS), Dysphagia Outcome and Severity Scale (DOSS), Oral transit time (OTT), Pharyngeal response time (PRT), Pharyngeal transit time (PTT), Airway closure time (AC), Upper oesophageal sphincter opening time (UES).

## Discussion

As far as we know, this is the first systematic review investigating the role of NIBS in enhancing motor outcomes in chronic stroke patients. We observed that specific motor functions, such as gait speed (10MWT), endurance (6MWT) [58,71], balance (TUG, CoP measures) [55,68], spasticity (MAS) [68], and lower limb movement (FM scale), showed greater improvements following tDCS treatment compared to sham. The predominant use of tDCS was also evident in studies combining RAT with NIBS: most of these studies [60,62,81,11, 83-85–] applied tDCS, while only one [82] used rTMS in conjunction with RAT for gait training. In contrast, upper limb motor functions, assessed using FM, MAL, ARAT, and MAS, tended to respond more favourably to rTMS than to sham treatment. However, when NIBS was combined with RAT for upper limb rehabilitation, tDCS was more commonly used; in fact, 4 out of 5 studies opted for tDCS over rTMS. A similar trend was observed for vocal and swallowing functional outcomes (e.g., Swallowing Quality of Life questionnaire – SWAL-QOL): the majority of studies included in this review (4 out of 6) administered rTMS, while the remaining two employed tDCS. Notably, only one of the tDCS studies combined the stimulation with conventional therapy [64].

Motor recovery after stroke is a challenging issue in the neurorehabilitation field [2]. It is well-known that functional recovery post-stroke in animal models occurs through the formation of new synaptic connections [88,89]. The surviving neurons in peri-lesional tissue show an enlargement of their dendritic trees as well as sprouting of axons to give birth to new connections in near and distant brain areas [90]. Classically, the driver of neuroplastic processes and hence functional recovery post stroke is training [91]. Aerobic exercise seems to be effective in releasing neurotrophins, such as brain-derived neurotrophic factor (BDNF) [92,93]. Recent research demonstrated that BDNF is involved in the neuroprotection, neurogenesis and neuroplasticity, and it has been identified as a key factor in motor learning and recovery after stroke [94]. Given this intimate relationship between neuroplasticity and motor performance, NIBS could represent a promising alternative/complementary treatment to improve stroke-induced deficits, directly manipulating brain excitability and plasticity [95].

It is worth noting that most stroke survivors present deficits in walking, upper limb activities, and swallowing functions. However, gait functions tend to recover easier than upper limb and swallowing functions [2, 96,97]. This could be explained by the fact that upper limb recovery is more closely tied to the integrity of the corticospinal tract (CST), a key neural pathway for voluntary motor control [2,98]. Accordingly, the majority of studies included in this systematic review have focused on upper limb recovery in patients with chronic stroke. The observed positive outcomes are likely associated with mechanisms such as cortical reorganisation, synaptic plasticity, and the structural and functional preservation of the CST [74,99].

The condition of the CST plays a crucial role in determining the effectiveness of interventions like rTMS. In patients with high CST integrity, low frequency rTMS targeting the contralesional hemisphere can reduce cortical hyperexcitability. This helps restore interhemispheric balance, supporting motor recovery [98]. On the other hand, in patients with low CST integrity, often associated with more severe motor deficits, high frequency rTMS applied to the contralesional hemisphere may support recovery by enhancing compensatory activity in the unaffected hemisphere [98,100]. This reflects a shift from the traditional interhemispheric inhibition model toward a compensation-based perspective. In this sense, recovery mechanisms may differ based on the degree of CST damage.

Additionally, axonal remodelling, such as CST sprouting and re-crossing from the contralesional hemisphere, has been identified as a key mechanism supporting upper limb recovery [98,101,102].

In the context of combining NIBS with robotic devices, such as exoskeletons and end-effectors, emerging evidence suggests a synergistic potential to enhance motor recovery. Robotic devices are a well-known strategy to boost neuroplasticity by providing intensive, repetitive, and task-specific training, while NIBS serves to further modulate cortical excitability and synaptic plasticity [2,23,103,104]

### tDCS

We found that the most used NIBS technique was the tDCS for restoring upper and lower limb outcomes. Relevant benefits provided by tDCS were registered for lower limb functions [68,69,73]. In particular, contra-lesional cerebellar tDCS showed promising results on balance recovery [55], whereas anodal tDCS over M1 promoted gait recovery [58,71]. However, the potential mechanisms underlying the improvement of gait performance induced by tDCS applied to M1-LL remain mainly speculative. An fMRI study on healthy subjects showed that four sessions of anodal tDCS applied to the lower limb area of the primary motor cortex (M1-LL) increased activation across a broad bilateral sensorimotor network, including the anterior cingulate gyrus, supplementary motor area (SMA), and somatosensory cortices [105]. This transient enhancement of sensorimotor network activity may underlie improvements in gait endurance and potentially contribute to increased gait speed.

This is further enhanced when the tDCS is combined with robotic gait training (RAGT), as suggested by other authors [60,11,84,85]. A recent systematic review [23] highlighted that tDCS applied to the leg area of the motor cortex in the affected hemisphere, or cerebellar tDCS targeting the ipsilesional or contralesional cerebellum in combination with RAGT, can lead to meaningful improvements in walking function. These improvements are especially evident in walking ability and capacity, as measured by tools such as the 6MWT.

The administration of tDCS in addition to RAGT suggests that this combined approach may promote spinal mechanisms of locomotion, such as spinal reflexes, central pattern generators and lower extremity electromyographic activity [23,56,106]. This approach could explain the rationale for the use of this combined approach. However, upper limb RAT combined with tDCS did not provide additional benefits to the only RAT, in three studies [61,79,80]. Specifically, Morone et al. [79] employed dual tDCS, combining inhibition of the unaffected hemisphere with excitation of the affected hemisphere. This approach is particularly recommended for patients with moderate to severe stroke, who may benefit from enhancing the compensatory role of the unaffected hemisphere. In contrast, for patients with milder stroke, single site tDCS targeting the affected hemisphere appears to be more effective in improving motor function [96].

Moreover, the effects of tDCS for the upper limbs included both sensory [107] and motor improvements [30,33,34,37,39,44,47,50,51]. Some authors [39,47] combined CIMT with brain stimulation, obtaining gains in grip strength and hand dexterity. Indeed, Rocha et al. [39] suggested that anodal tDCS seems to have a greater impact on upper limb motor outcomes than cathodal tDCS.

On the other hand, positive effects of tDCS on vocal and speech functions were also found, as suggested by Wong et al. [59]. The authors applied a combined intervention of speech therapy and tDCS to enhance outcomes in dysarthric speech. Similarly, Ahn et al. [64] reported positive effects of tDCS paired with conventional therapy in improving swallowing function in patients with dysphagia.

### i-TBS

i-TBS demonstrated its efficacy on the recovery of gait and balance functions, especially when the stimulation was applied to cerebellar lobes [70]. Indeed, Koch et al. [73] demonstrated that i-TBS was effective in improving not only gait and balance functions, but also ankle stability of the paretic lower limb. However, Lin et al. [74] concluded that applying i-TBS to bilateral lower extremity (LE) M1 was not effective in enhancing lower limb functions. The authors suggested that bilateral stimulation may be appropriate when motor evoked potentials (MEPs) from the affected hemisphere are unrecordable, as it ensures that the induced current reaches both motor cortices. However, when MEPs from the affected hemisphere are present, unilateral stimulation targeting the affected LE M1 may be more effective.

Moreover, the i-TBS was also administered to improve upper limb functions. According to findings of the included studies, it seems to be effective in reducing upper limb spasticity [36] and in modulating corticospinal excitability [40] when it is applied alone. However, Sung et al. [42] and Di Lazzaro et al. [43] administered iTBS joined with rTMS, suggesting that this kind of stimulation is safe and promotes motor recovery in chronic stroke patients. Zhang et al. [78] investigated the effects of both priming and non-priming iTBS combined with RAT for upper limb rehabilitation. They found that priming iTBS, specifically, applying continuous TBS before iTBS, was more effective in patients with relatively preserved upper limb function compared to non-priming approaches. The authors hypothesized that priming iTBS may enhance motor learning by increasing the responsiveness of the ipsilesional supplementary motor area to therapeutic inputs, such as mirror visual feedback. However, Talelli et al. [67] reported no additional benefit of TBS for improving upper limb and hand function. They emphasized the importance of patient selection, noting that attempting to promote ipsilesional reorganization may be unrealistic in individuals with severe brain damage.

### rTMS

Lastly, rTMS was particularly used to improve upper limb functions [31,32,35,38,41–43,45,53]. Some authors [32,35,41,48] suggested its use to reduce spasticity by increasing the unaffected hemisphere’s excitability and decreasing spinal excitability. Similarly, Rastgoo et al. [75] found improvements in lower limb muscle spasticity but using low frequency rTMS. However, Etoh et al. [49] did not register any changes in spasticity after rTMS, despite finding gains in motor functions. According to the authors’ conclusion [49], this finding is linked to the limited ability of the MAS to detect small changes in spasticity, as it primarily identifies only more significant improvements. Despite being the most widely used clinical scale for assessing spasticity, the MAS may not capture subtle clinical changes [108]. The authors hypothesise that improvements in spasticity are more evident in patients with a severe MAS score.

Moreover, we found some evidence [75–77] about rTMS in the recovery of the lower limb in chronic post-stroke patients. According to literature [75–77,82], rTMS promoted functional recovery of lower limb and gait functions. Specifically, Wang et al. [77,82] found that rTMS alone [77] and/or combined with treadmill training [82] had positive effects on gait speed and spatial symmetry.

On the other hand, rTMS was used to improve swallowing functions [65,66,86,87], reducing risks of aspiration in patients with chronic post-stroke. However, only Cheng et al. [66] found positive effects of excitatory rTMS on swallowing post-stroke symptoms. Other authors [65,86] did not found any improvement in dysphagic symptoms, which may be related to their study design and severity of stroke. The rTMS for dysphagic symptoms should be applied on the unaffected hemisphere, considering also that patients with severe stroke may have smaller improvements than moderate and mild stroke.

### Comparative analysis of the different NIBS techniques

Among the various NIBS techniques used in chronic stroke rehabilitation, tDCS was the most widely applied, particularly for enhancing both upper [30,33,34,37,39,40,44,48,50–53] lower limb [55,56,58,60,62,68,69,71,81,11, 83–85] motor functions. tDCS showed significant benefits for lower limb recovery, with contra-lesional cerebellar stimulation improving balance [55] and anodal stimulation over the M1 promoting gait function [23]. These effects are further enhanced when it is combined with RAGT [11,60,62,81,83,84,85]. Additionally, tDCS has been linked to improvements in upper limb strength and dexterity, particularly when paired with constraint-induced movement therapy [47], and it showed promise for treating speech and swallowing disorders [59,64,86]. The advantages of tDCS include its simplicity, low cost, portability, and ease of integration with physical therapies, making it a widely accessible option for post-stroke rehabilitation. However, a significant limitation of current tDCS protocols is their reliance on fixed stimulation parameters, such as electrode placement, stimulation intensity, and polarity (anodal vs. cathodal). These factors may not account for individual differences in neurophysiological responses. Future research could benefit from a more tailored approach, adjusting tDCS to preserve critical neural pathways, such as the CST, and guiding it with MEPs. This individualised approach, where both stimulation parameters and electrode sites are customised to each patient’s unique brain activity, could enhance tDCS efficacy and lead to more consistent improvements in motor function. Furthermore, in the future, neuro-navigation and real-time EEG monitoring of NIBS effects may help reduce variability in outcomes, offering a more precise and personalized treatment method.

Moreover, iTBS has demonstrated promise in recovering gait, balance, and lower limb stability [70,72–74], especially when applied over the cerebellar lobes. It also reduces upper limb spasticity [36] and modulates corticospinal excitability, with more notable outcomes when used with priming protocols or in combination with other NIBS approaches like rTMS [42,43]. However, iTBS requires highly accurate targeting and appears to be more effective in patients with preserved motor function, limiting its use in more severe cases.

rTMS is primarily used to support upper limb motor recovery and reduce spasticity, often through low-frequency stimulation of the unaffected hemisphere [31,32,38,40–43,45,49,53,54]. It has also shown potential benefits in lower limb function and gait symmetry [75–77], particularly when combined with treadmill training [82]. However, the role of rTMS in swallowing rehabilitation remains inconclusive due to mixed study results [65,66,86,87]. rTMS is valued for its precise cortical targeting and well-established protocols, but it is more expensive, less accessible, and less portable than other NIBS techniques. Additionally, its efficacy may vary depending on stroke severity and stimulation parameters.

Although overall results showed clinical benefits across all NIBS modalities, differences in underlying mechanisms may partly explain the variability in treatment response. tDCS modulates cortical excitability through subthreshold changes in neuronal membrane potentials, leading to long-term neuroplastic effects when combined with task-specific training [12,13]. In contrast, rTMS induces direct neuronal depolarization via magnetic pulses, allowing for more focal and frequency-dependent modulation of cortical circuits [1,3,4]. These mechanistic distinctions may explain the stronger or more consistent effects reported with rTMS in some motor outcomes [2,5]. However, individual variability, stimulation parameters, and integration with rehabilitation protocols also contribute to treatment efficacy [13–15].

In summary, while each NIBS technique offers distinct benefits, their effectiveness depends on patient-specific factors such as stroke severity, residual function, and therapy goals (see Table 5) [39,45,55,73].

**Table 5:** Summary of advantages and disadvantages of each NIBS techniques, according to the literature.

Technique	Advantages	Disadvantages
**tDCS**	• Low cost and easy to administer. • Portable and well-tolerated. • It can be easily combined with physical/robotic therapies. • Shown effective for motor (UL and LL), speech, and swallowing recovery.	• Limited spatial precision. • Effects highly variable between individuals. • Mechanisms not fully understood. • Limited efficacy in severely damaged cortices.
**iTBS**	• Short application time. • Effective for gait, balance, and spasticity (especially cerebellar stimulation). • Can be used with priming or other NIBS methods.	• Requires accurate targeting. • Less effective in patients with severe impairments • Mixed results for upper limb recovery. • Limited evidence for speech/swallowing benefits.
**rTMS**	• Precise cortical targeting. • Well-established in motor recovery (esp. upper limb). • Reduces spasticity and promotes gait improvements (when combined with training).	• Expensive and less accessible. • Bulky equipment, not portable. • Variable effectiveness depending on stroke severity. • Inconsistent results in swallowing recovery.

The best outcomes are often achieved through individualized, combined approaches that leverage the strengths of each technique. The choice of intervention should be tailored to the patient’s clinical profile and the targeted functional outcomes, with the understanding that a personalised, multi-modal approach may yield the most significant benefits in post-stroke rehabilitation.

### Strengths and limitations

This review presents a comprehensive and qualitative synthesis of the current evidence on the effectiveness of NIBS in the field of chronic stroke. It highlights the potential of NIBS interventions to promote motor functional recovery, in addition to conventional or robotic-assisted therapy, fostering neuroplastic changes. The strengths of this review lie in its analysis of diverse applications of NIBS, providing insights into its therapeutic possibilities. Additionally, the inclusion of robotic devices in addition to neuromodulation therapy reflects the growing emphasis on personalised treatment approaches.

However, several limitations must be acknowledged. This review is based solely on a qualitative analysis, which, while providing valuable descriptive insights, does not allow for statistical evaluation of effect sizes or direct comparisons between studies. As a result, our review provided a comprehensive qualitative synthesis of the available evidence, offering valuable insights into the field of NIBS applications for specific motor deficits in individuals with chronic stroke, identifying key implications for clinical practice and considerations for future investigation.

Despite the large number of selected studies, many included trials were conducted with small sample sizes and heterogeneous methodologies, limiting the generalizability of the findings. Furthermore, the lack of standardised NIBS protocols complicates comparisons across studies. These limitations pertain to the quality and design of the included studies and should be considered when interpreting the results.

Limitations of the review process itself must also be acknowledged. Although rigorous methods were applied, including blinded screening and inter-rater agreement assessment, the possibility of publication bias and language bias cannot be fully excluded, as only studies published in English and peer-reviewed journals were considered.

Several questions remain open, such as the optimal NIBS dosage, the most effective timing of administration (e.g., before or after rehabilitation therapy), and the duration of its retained effects. Future research should prioritise longitudinal clinical trials to assess whether the benefits of NIBS are sustained over extended periods. Given the persistent nature of motor deficits, it is essential to determine if NIBS-induced motor recovery remains effective over months or years, thus providing stronger evidence for its integration into long-term rehabilitation management strategies.

## Conclusion

In conclusion, this review offers a comprehensive overview of the role of NIBS in enhancing motor outcomes for chronic stroke patients, with a focus on both upper and lower limb as well as swallowing function recovery. Specifically, NIBS techniques such as tDCS, iTBS, and rTMS have been shown to improve gait speed (10MWT), endurance (6MWT), balance (TUG test), and spasticity (MAS). For upper limbs, improvements in motor function were observed across multiple scales, including the FM, MAL, and ARAT, particularly with rTMS and tDCS when combined with rehabilitation therapies like CIMT. However, the results across studies are inconsistent, which can be attributed to several factors. These include variations in stroke severity, individual neurophysiological responses to stimulation, and methodological differences such as sample sizes and protocols. Despite the abundance of literature and well-designed RCTs, the results of the studies selected are not entirely consistent with each other. Furthermore, there is still limited evidence regarding the efficacy of NIBS on vocal and swallowing symptom recovery after chronic stroke.

To address these inconsistencies, future research should prioritize the development of more personalized, tailored NIBS protocols that account for individual differences in brain activity and stroke characteristics. For example, adjusting stimulation parameters based on MEPs and targeting critical neural pathways like the CST may optimize outcomes. Additionally, integrating real-time EEG monitoring and neuro-navigation technologies could enhance the precision of NIBS application and reduce variability in treatment effects.

This review aligns with existing research by reaffirming the potential of NIBS as a promising intervention for chronic stroke rehabilitation. However, it also challenges current approaches by emphasizing the need for individualized treatment strategies rather than fixed stimulation parameters. Given the variability in patient responses, a more customized, multimodal approach combining NIBS with physical therapies such as robotic-assisted training may yield more consistent and significant improvements in motor function.

In summary, while NIBS show substantial promise, further investigations are needed to refine protocols, understand underlying mechanisms, and establish long-term effectiveness. These steps will be crucial for integrating NIBS into routine clinical practice for stroke rehabilitation. Future studies should also optimize training protocols considering the clinical and personal features of patients who may benefit most from these promising treatments.

## Supporting information

S1 Table RoB of all included studies. (XLSX)

S2 Table Table with excluded papers. (XLSX)

S3 Table Table with included papers. (XLSX)

S1 File PRISMA 2020 checklist. (PDF)
